# Allele Frequency Difference *AFD*–An Intuitive Alternative to *F_ST_* for Quantifying Genetic Population Differentiation

**DOI:** 10.3390/genes10040308

**Published:** 2019-04-18

**Authors:** Daniel Berner

**Affiliations:** Department of Environmental Sciences, Zoology, University of Basel, Vesalgasse 1, CH-4051 Basel, Switzerland; daniel.berner@unibas.ch; Tel.: +41-(0)-61-207-03-28

**Keywords:** genetic differentiation, minor allele frequency, population genetics, sample size, single-nucleotide polymorphism

## Abstract

Measuring the magnitude of differentiation between populations based on genetic markers is commonplace in ecology, evolution, and conservation biology. The predominant differentiation metric used for this purpose is *F_ST_*. Based on a qualitative survey, numerical analyses, simulations, and empirical data, I here argue that *F_ST_* does not express the relationship to allele frequency differentiation between populations generally considered interpretable and desirable by researchers. In particular, *F_ST_* (1) has low sensitivity when population differentiation is weak, (2) is contingent on the minor allele frequency across the populations, (3) can be strongly affected by asymmetry in sample sizes, and (4) can differ greatly among the available estimators. Together, these features can complicate pattern recognition and interpretation in population genetic and genomic analysis, as illustrated by empirical examples, and overall compromise the comparability of population differentiation among markers and study systems. I argue that a simple differentiation metric displaying intuitive properties, the absolute allele frequency difference *AFD*, provides a valuable alternative to *F_ST_*. I provide a general definition of *AFD* applicable to both bi- and multi-allelic markers and conclude by making recommendations on the sample sizes needed to achieve robust differentiation estimates using *AFD*.

## 1. Introduction

Biological studies measuring the magnitude of genetic differentiation between populations, for example to explore levels of gene flow between populations, to discover genome regions influenced by natural selection, or to inform decisions in conservation biology, are published on a daily basis. A differentiation metric used frequently in such work is *F_ST_*, interpreted broadly as a measure of the proportion of the total genetic variation at a genetic locus attributable to differentiation in allele frequencies between populations [1]. *F_ST_* was conceptualized in the middle of the last century as a descriptor of genetic structure among populations [2,3,4]. Over the subsequent decades, numerous estimators were developed to allow *F_ST_* to be calculated with empirical genetic data, based on different assumptions about the sampled study populations and/or the mutation process of the genetic markers [3,5,6,7,8,9,10,11,12,13,14]. Aside from some controversy about how to best calculate *F_ST_* with multi-allelic genetic markers such as microsatellites [14,15,16,17,18,19,20,21], the fundamental concept shared among the *F_ST_* estimators is firmly established in population genetics and genomics; *F_ST_* is currently among the most widely used statistics in these fields. In this note, I will argue that despite its popularity, *F_ST_* has shortcomings that complicate the analysis of population differentiation, and that a powerful alternative differentiation metric is available.

## 2. Features of an Appropriate Differentiation Metric

To approach the problems inherent in *F_ST_*, we will start from the very beginning and ask what properties a metric of genetic differentiation should exhibit. First, the scale of the metric should range from zero (no genetic differentiation among populations) to one (complete fixation for different alleles). This familiar scale greatly facilitates interpretation and allows for convenient comparisons of differentiation among genetic markers and study systems. *F_ST_* estimators satisfy this scale criterion; they are generally designed to range from zero to one.

**Figure 1:**
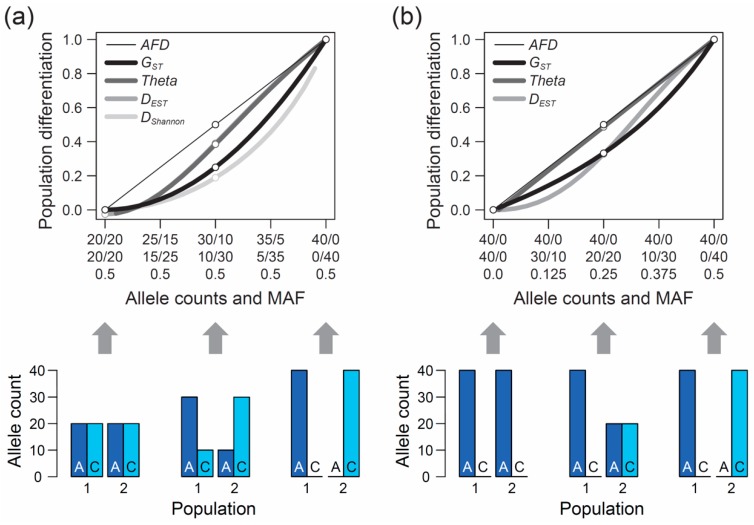
Population differentiation expressed by different metrics. Magnitude of genetic differentiation at a bi-allelic single-nucleotide polymorphism (SNP) along the continuum of allele frequency differentiation between two populations (top graphs). Differentiation is quantified by the absolute allele frequency difference (*AFD*), by two popular estimators of *F_ST_* (*G_ST_* and *Theta*), by *D_EST_*, and by Shannon differentiation (*D_Shannon_*). The X-axis specifies the underlying allele counts in population 1 (first row) and population 2 (second row) for two hypothetical alleles (A, C), assuming a draw of 40 total alleles per population at the exact allele frequencies in each population (no sampling stochasticity). The third row gives the frequency of the less common SNP allele across the pool of the two population samples (i.e., the pooled minor allele frequency, MAF). The SNP is specified to exhibit a maximal MAF in (**a**) and a minimal MAF in (**b**) (for the latter, *D_Shannon_* is undefined mathematically). The bar plots on the bottom illustrate the counts of the two alleles for three levels of differentiation (none, intermediate, complete). Note that some metrics are undefined at the endpoints of the differentiation continuum, and that in (**a**), *D_EST_* very closely approximates *Theta* and is therefore hidden.

The second, perhaps even more crucial requirement of an appropriate differentiation metric is that it should show an intuitive and traceable relationship to the magnitude of genetic differentiation between populations, so a researcher can understand and interpret what they are measuring. But what should this relationship look like? The answer to this question cannot be derived from theory but depends on the needs and expectations of the researchers measuring differentiation among their study populations. To develop a sense for these expectations, I performed a qualitative survey involving a total of 15 haphazardly chosen colleague researchers (advanced postdocs and faculties) having years of experience in population genetics and/or evolutionary genomics, including both empiricists and theoreticians. I confronted these researchers with a graphic displaying a continuum of symmetrically increasing genetic differentiation between samples of nucleotides (n = 40) drawn from two hypothetical populations at a single-nucleotide polymorphism (SNP) (X-axis, ranging from no to complete differentiation). I then asked them to specify the corresponding magnitude of population differentiation (*Y*-axis) an ideal metric of differentiation should exhibit if such a metric was to be invented from scratch. Specifically, the respondents were presented in Figure 1a, with the upper panel of the figure left blank.

Although considered a qualitative rather than formal investigation, and despite a modest sample size, this survey produced a clear result: among the 15 total researchers, 13 argued that the most intuitive differentiation metric would exhibit a linear relationship from zero to one along this continuum of allele frequency shifts between populations, as shown by the thin black line in Figure 1a. Exactly this relationship is expressed by the absolute allele frequency difference, hereafter *AFD*. For a single bi-allelic marker, *AFD* is easily obtained by arbitrarily defining one of the two alleles as the focal allele and calculating the absolute difference in the frequency of this allele between the populations. (‘Allele proportion’ would perhaps be a more precise term than ‘allele frequency’, but I will stick to the latter expression used traditionally in population genetics.) More generally, the calculation of *AFD* between two populations at a genetic polymorphism can be formalized as

$$AFD=\frac{1}{2}\overset{n}{\underset{i=1}{\sum }}\left|\left(f_{i1}-f_{i2}\right)\right|$$

where *n* represents the total number of different alleles observed at the polymorphism, and the *f_i_*-terms specify the frequency of allele *i* in the two populations (an analogous definition is given verbally in Reference [22]). This formula can also be applied to multi-allelic markers like microsatellites. The focus of this paper, however, lies on standard bi-allelic SNPs, given that this type of polymorphism has become the predominant genetic marker. A worked example of *AFD* calculation for both a bi-allelic SNP and a multi-allelic microsatellite is provided as Analysis S1 in the Supplementary Materials (for applications of *AFD* in recent genomic investigations see References [23,24,25,26,27]).

## 3. Some Problems with *F_ST_*

As suggested above, a substantial proportion of researchers appear to find the linear relationship to continuous genetic differentiation exhibited by *AFD* particularly intuitive and interpretable. Note that throughout this paper, (non-)linearity refers only to the immediate relationship of a given differentiation metric to population allele frequencies, and hence does not imply any specific relationship of the estimator to biological factors influencing allele frequencies, such as gene flow, mutation, selection, population size, or divergence time. Now let us consider how *F_ST_* behaves along the continuum of differentiation in allele frequencies. For this, we will initially focus on the two most popular *F_ST_* estimators, *G_ST_* [6] and *Theta* (*θ*) [8] (given in more accessible notation by the Formulas (8) and (6) in Reference [28]) and consider other metrics later. I emphasize that the insights emerging from these explorations may not be novel to researchers closely familiar with the theory underlying *F_ST_*, but they are clearly under-appreciated by empiricists.

When the populations are undifferentiated genetically, *G_ST_* is zero, as one would expect (Figure 1a, left end on the X-axis). Likewise, if the two population samples are monomorphic for alternative alleles, differentiation is at its maximum and *G_ST_* exhibits the intuitive value of one (Figure 1a, right end on the X-axis). Between these extremes, however, the relationship between allele frequency change and *G_ST_* is non-linear. Specifically, within the domain of low population differentiation, a unit increase in the frequency of the allele *A* in population 1 and a corresponding increase in the frequency of *C* in population 2 causes a negligible increase in *G_ST_*. A similar unit allele frequency change, however, drives a disproportionally large increase in *G_ST_* when the populations are close to complete differentiation (Figure 1a). *Theta* shows qualitatively similar properties, although the deviation from *AFD* is less pronounced than for *G_ST_*, except when differentiation is very weak.

The above numerical investigation assumes that the frequency of the minor (or less common) allele, as determined based on the *pool* of the two populations (hereafter MAF, for minor allele frequency), is maximal (i.e., 0.5 across the entire population differentiation range). It is instructive to also explore the behavior of our focal differentiation metrics when the MAF is minimal (i.e., one population is consistently monomorphic). Under this condition, the relationship between genetic differentiation and *AFD* remains straightforward to interpret. For instance, an allele frequency differentiation exactly intermediate between the absence of differentiation (i.e., both populations are monomorphic for the same allele) and the complete fixation for alternative alleles between the populations still yields an intuitive *AFD* value of 0.5 (Figure 1b). Reducing the MAF, however, has a strong influence on the *F_ST_* estimators; their deviation from *AFD* declines. In particular, *Theta* now essentially coincides with *AFD* (the deviation of *Theta* from *AFD* under the full range of allele frequency combinations between the two populations is presented in Figure S1a).

**Figure 2:**
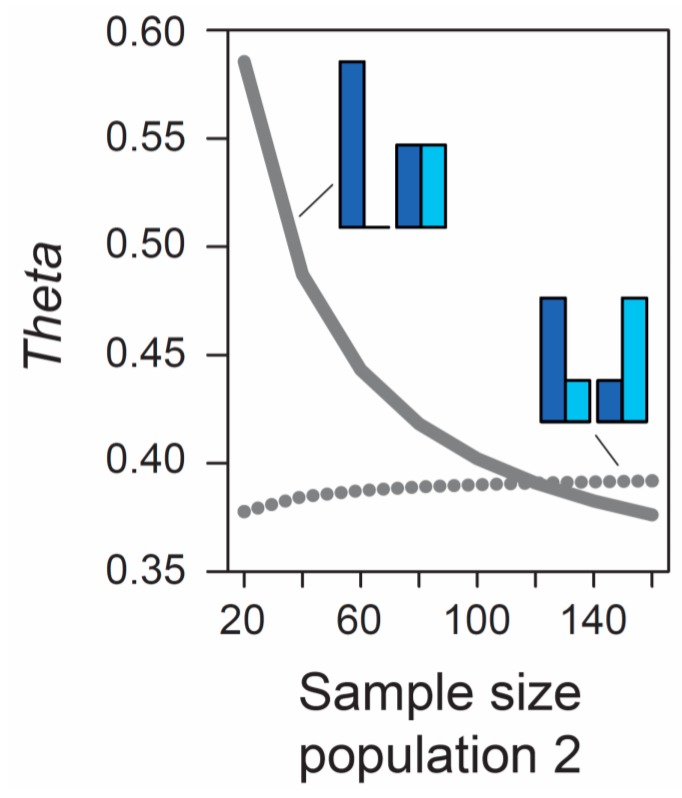
Influence of sample size imbalance between populations on the magnitude of the *F_ST_* estimator *Theta* at a single SNP. Sample size for population 1 is always 40 nucleotides, as in Figure 1, but sample size for population 2 varies from 20 to 160 nucleotides (X-axis). Two different MAF levels are considered (minimal, solid line; maximal, dotted line). Sampling occurs deterministically at the exact population allele frequencies illustrated by the bar plots. For both MAF levels and across the full parameter range considered, the magnitude of allele frequency differentiation between the populations is therefore invariably perfectly intermediate (*AFD* is 0.5 throughout).

Beside the MAF, the influence of sample size on metrics of differentiation also deserves attention. The formulas underlying the calculation of *AFD* and *G_ST_* rely exclusively on allele frequencies and thus ignore the sample sizes used to estimate these frequencies. Therefore, the expected (parametric) value of these differentiation metrics is not dependent on sample size. (Empirical values derived from stochastic real-world samples, however, will be influenced by the precision underlying the estimation of allele frequencies, and hence by sample size, as elaborated in a separate section below). By contrast, the expected value of *Theta* at a given marker does depend on sample size. As long as sample sizes are similar between the two focal populations, the absolute size of these samples has a relatively minor influence on *Theta*, at least for typical (not very small) sample sizes used by empiricists (details not presented). However, imbalance in the size of the samples from the populations can have a dramatic influence on *Theta*. To appreciate this point, we assume that we sample nucleotides at a genetic marker from two populations exhibiting intermediate differentiation in allele frequencies (*AFD* = 0.5). Sample size for the first population is always constant (n = 40 nucleotides, as in Figure 1), whereas sample size for the second population is variable, ranging from 20 to 160 nucleotides. If the MAF is chosen to be minimal, we observe a dramatic decline in *Theta* as sample size for the second population increases (solid line in Figure 2). For example, all else equal, *Theta* declines from 0.59 to 0.42 when increasing sample size for the second population from 40 to 80 (equivalent to 20 and 40 diploid individuals). By contrast, choosing allele frequencies in the two populations such that the MAF is maximal, we find that the influence on *Theta* of sample size imbalance between the populations is reversed in direction, and weaker in magnitude (dotted line in Figure 2). Note that these effects are unrelated to sampling stochasticity, as we assume that our samples always mirror exactly the true population frequency (as in Figure 1).

Collectively, the above explorations allow us to draw a number of important conclusions regarding *F_ST_*. First, *F_ST_* generally displays a non-linear relationship to continuous population differentiation in allele frequencies (note that *G_ST_* is sometimes claimed to display a perfectly linear relationship, and thus to coincide with *AFD*, after square-root transformation. This view is incorrect, as demonstrated in Figure S1b). This non-linearity has a more serious implication than just being unintuitive to many scientists: in several research fields using marker-based inference, small differences in the magnitude of genetic differentiation between population comparisons are highly relevant—and yet, this is exactly the domain in which *F_ST_* is least sensitive (Figure 1a,b). For instance, observing average *AFD* of 0.05 versus 0.1 in two different population comparisons may point to an interesting difference between these population pairs in the opportunity for gene flow. However, when expressed as *F_ST_*, the corresponding difference in the average magnitude of differentiation between the two population comparisons may appear marginal and not attract a researcher’s attention. *F_ST_* thus compromises the comparability of differentiation among markers and among studies in the differentiation range most interesting to many empiricists. This point is reinforced by two investigators involved in the above survey having argued that although a linear increase in a differentiation metric along the range of continuous genetic differentiation appears ideal, one could also imagine a non-linear relationship with *elevated* sensitivity across the lower population differentiation range. *F_ST_* behaves exactly opposite to this suggestion.

Our second conclusion is that genetic differentiation values expressed by *F_ST_* are contingent on the MAF of the population system (see also References [29,30,31]). While there may be reasons justifying this behavior in specific analytical contexts, it will appear unintuitive (and often be unknown) to empiricists that a given magnitude of allele frequency differentiation yields different *F_ST_* values depending on how balanced the two alleles are in the population pool.

The third conclusion is that at least some *F_ST_* estimators, like *Theta*, are sensitive to the balance between populations in the number of nucleotides sampled at a given SNP. This property must be considered a serious nuisance to empiricists, especially those working with high-throughput sequencing data from population pools [32,33]. Even when controlling the number of study individuals in a population genomic experiment tightly, high-throughput sequencers inevitably generate variation in read depth among genomic positions, which will systematically inflate the variance in *F_ST_* among markers and among studies when using *Theta* as estimator. Moreover, that the *expected* value of differentiation at a marker should shift when the samples drawn from the focal populations differ in size—even when the allele frequency estimates remain exactly the same—and that this influence of sample size imbalance on *F_ST_* is itself contingent on the MAF, can hardly be considered intuitive.

## 4. Other Differentiation Estimators

So far, our reflections on *F_ST_* were based on the commonly used estimators *G_ST_* and *Theta*. A number of less widely applied alternative *F_ST_* estimators have been introduced, however, hence it is valuable to examine if these estimators share with the former the weaknesses identified above. For this, I repeated the analysis of the magnitude of differentiation along the two continua of differentiation in allele frequencies visualized in Figure 1 with Wright’s *F_ST_* [3,5] (Formula (1) in Reference [16]) and *Phi_ST_* (*Φ*) [10]. For the case of a bi-allelic SNP considered here, these metrics yielded values identical to *G_ST_*. Similarly, repeating the calculations with Hudson’s *F_ST_* [11] (Formula (10) in Reference [28]) produced results identical to *Theta*. Clearly, the problems identified for *G_ST_* and *Theta* extend to the whole family of *F_ST_* estimators. Moreover, *D_EST_* (Formula (13) in Reference [14]), proposed as an alternative or complement to *F_ST_*, exhibited differentiation values qualitatively similar to the *F_ST_* estimators (Figure 1; see also References [34,35]).

Overall, the complexity inherent in *F_ST_* (non-linearity, MAF- and sample size-dependence, difference among estimators) makes clear that *F_ST_* was not designed as a simple descriptor of allele frequency differentiation. Instead, *F_ST_* estimators aim at quantifying progress in population differentiation, or at partitioning genetic variation among hierarchical levels, in the light of specific models of mutation, gene flow, and drift [3,5,11,36,37,38,39,40]. Empiricists using *F_ST_*, however, will rarely be aware of the underlying assumptions, and even for those who are, real-world situations will generally not allow evaluating what—if any—evolutionary model is meaningful for any given population pair, genome region, and marker analyzed (for a similar view see Reference [37]). It is therefore not surprising that the question of how *F_ST_* should best be interpreted, and what constitutes the optimal *F_ST_* estimator in the first place, has been a matter of debate for decades [1,14,16,20,21,29,34,37,40,41,42]. To conclude, it is not generally clear what quantity *F_ST_* measures in empirical contexts—a view in line with the wide-spread sentiment of researchers that an intuitive differentiation metric should behave differently from *F_ST_*. Replacing or complementing the complex theory-laden *F_ST_* differentiation metrics by a traceable descriptor of differentiation independent from any specific population genetic model thus promises to facilitate the identification and interpretation of patterns in population differentiation, and to increase the comparability among studies and markers. The simple absolute allele frequency difference *AFD* appears adequate for this purpose.

As a potential alternative to *AFD*, I further considered a metric derived from information theory called Shannon differentiation (hereafter *D_Shannon_*) that has recently been claimed to exhibit a ‘straightforward relationship to allele frequency differences’ [43] (see also References [44,45]). I explored how this novel metric behaves across the continua of allele frequency differentiation, which revealed two major shortcomings: first, *D_Shannon_* exhibits even less sensitivity than *F_ST_* in the domain of weak to modest allele frequency differentiation between populations (Figure 1a). Consequently, *D_Shannon_* deviates even more strongly from the relationship to allele frequency differentiation considered desirable by many investigators. Second, *D_Shannon_* is undefined mathematically as soon as one population is monomorphic, that is, fixed for one allele (hence *D_Shannon_* is undefined across the entire differentiation continuum in Figure 1b). Given these problems, it appears doubtful that *D_Shannon_* will generally be considered a valuable differentiation metric and adopted widely for empirical analysis.

The latter conclusion also applies to ad hoc differentiation metrics based on *p*-values derived from statistical tests of differentiation between populations at genetic markers (e.g., References [46]). The disadvantage of such metrics is that we generally cannot easily translate a locus-specific *p*-value (i.e., the probability of an observed effect size) quantitatively into progress toward complete genetic differentiation (the effect size itself). In addition, *p*-values are a direct function of sample size, further reducing the comparability among markers and studies.

## 5. *F_ST_* Can Complicate or Mislead the Biological Interpretation of Differentiation Data—Two Examples

In the previous section, the drawbacks of *F_ST_* (and related metrics) were exposed based on simple numerical analyses. Given the ubiquity of *F_ST_* in empirical research, I next illustrate implications of quantifying population differentiation by *F_ST_* in real-world genetic analyses based on two examples from threespine stickleback fish (*Gasterosteus aculeatus* L.).

**Figure 3:**
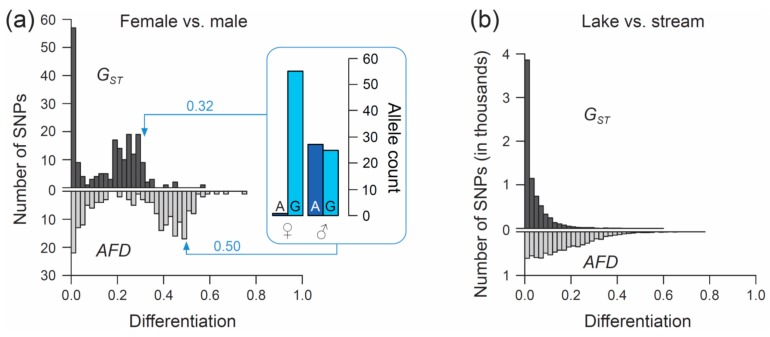
Performance of *F_ST_* versus *AFD* in empirical analyses. (**a**) Distribution of the magnitude of differentiation, as measured by the *F_ST_* estimator *G_ST_* (upper histogram) and by *AFD* (lower histogram), between female and male threespine stickleback across 200 SNPs located on chromosome 19. The box visualizes the sex-specific allele counts at one exemplary SNP representative of the upper mode of each distribution, with *G_ST_* and *AFD* for this marker given next to the arrows. (**b**) Distribution of *G_ST_* and *AFD* values across 7282 genome-wide SNPs in a lake and stream stickleback population comparison.

The first example re-uses SNP data generated through individual-level RAD sequencing (based on *Sbf1* enzyme restriction) in 28 female and 26 male stickleback from a single population inhabiting Misty Lake, Vancouver Island, Canada (the pooled lake and outlet samples from Reference [47]; for background information on this population see References [48,49,50]). We focus exclusively on SNPs (n = 200) located on chromosome 19, and we ask what distribution (visualized by a simple histogram) the differentiation between the sexes at SNPs along this chromosome will exhibit when quantified by the *F_ST_* estimator *G_ST_*, and by *AFD*. Our key observation is that both metrics indicate a bi-modal distribution of differentiation values, but that the high-differentiation mode is located in a lower differentiation range for *G_ST_* (upper mode at around 0.25–0.3) than for *AFD* (upper mode near 0.5) (Figure 3a). The analytical relevance of this difference in the distribution of differentiation values becomes clear when considering that the threespine stickleback has a chromosomal XY sex determination system, and that the focal chromosome 19 represents the sex chromosome [51]. Crossover between the X and Y gametologs is restricted to a short segment of chromosome 19 [52,53]. Across the rest of the chromosome, the two gametologs represent completely isolated and deeply divergent populations. Consequently, the X and Y have reached (or are close to) fixation for distinct alleles at numerous SNPs. These gametolog-distinctive alleles cause the high-differentiation mode in both histograms, because the females (XX) are homozygous while the males (XY) are heterozygous at these SNPs (confirmed by inspecting allele frequencies in females and males at ten haphazardly chosen SNPs exhibiting *AFD* near 0.5; one example is presented within the box in Figure 3a). In other words, for any SNP allele private to the X, females tend to display a 100% frequency while males display a 50% frequency (note that SNPs with low male read coverage, indicating alignment problems for the Y-derived sequences, were excluded). Obviously, an intuitive differentiation metric—that is, a metric facilitating the understanding of the link between the magnitude of differentiation and the underlying biological cause—should yield a value of 0.5 for such a marker. While *AFD* shows this property, *G_ST_* clearly impedes biological interpretation; to understand that the location of the upper differentiation mode in *G_ST_* indicates a high abundance of SNPs with (nearly) X- and Y-limited alleles, one needs to be aware of the specific function linking allele frequency differences to *G_ST_* (Figure 1a,b). Note that due to the peculiar allele distribution between the sexes, causing the MAF across the pool of the sexes to be minimal, *Theta* fortuitously approximates *AFD* in this specific empirical example (details not presented).

For the second example, I re-use SNP data from a young stickleback population pair inhabiting ecologically different but adjacent lake and stream habitats in the Lake Constance basin in Central Europe. Lake and stream stickleback in the Lake Constance basin occupy different foraging niches [54,55], are under divergent natural selection (as revealed by both marker-based divergence mapping and transplant experiments [56,57]), and show partial sexually based reproductive barriers [58]. We here perform a genetic comparison between the Lake Constance (n = 25 individuals) and the BOH stream (n = 22) population pair from Reference [56] (see also Reference [54]) and examine the distribution of both *G_ST_* and *AFD* across genome-wide SNPs. For this, the original raw marker data set (generated by RAD sequencing based on *Nsi1* enzyme restriction; see Reference [56] for details) was subject to the following filters: first, I considered only bi-allelic SNPs represented by at least 32 nucleotides in each population. Second, a SNP was accepted only when located at least 12 nucleotide positions away from its nearest SNP, thus effectively excluding pseudo-SNPs caused by micro-indels within a RAD locus. Finally, only SNPs exhibiting a MAF of at least 0.45 across the population pool were considered. This latter filter dramatically reduced the data set (7282 SNPs remaining), but ensured that only those SNPs having the potential to span almost the full differentiation range entered analysis (i.e., markers with nearly maximal information content *sensu* [59]; markers with a low MAF are constrained to produce low differentiation values and thus bias the distribution of differentiation values, see also Reference [60]).

Comparing the genome scans in the lake-stream stickleback pair performed with *G_ST_* and *AFD* as differentiation metrics reveals an important difference: the distribution of SNP-specific *G_ST_* values has a striking mode near zero, tapering off steeply into a thin tail of higher differentiation values (strongly ‘L-shaped’ distribution, Figure 3b; see also Figure 3 in Reference [57]), whereas the *AFD* distribution is more uniform. Likewise, summary point estimates of differentiation differ substantially between the two metrics: genome-wide median *G_ST_* is only around 0.02 whereas median *AFD* reaches 0.13, and the highest-differentiation SNP scores only 0.59 with *G_ST_* but 0.77 with *AFD* (a scatterplot showing *F_ST_* against *AFD* across all SNPs for this population comparisons is presented in Figure S2). These differences in the distribution of differentiation values between the metrics are important because they may stimulate qualitatively different biological interpretations: the *F_ST_* distribution would commonly be taken as evidence that most of the genome is homogenized by gene flow between the adjoining populations, with substantial differentiation maintained by strong divergent selection in a few genome regions only [61,62]. But is such a mechanistic interpretation justified? A more cautious view is that for purely mathematical reasons (i.e., the lack of sensitivity in the low-differentiation domain), *F_ST_* estimators will return a strongly L-shaped differentiation distribution for any population pair exhibiting weak differentiation—no matter what combination of evolutionary processes this differentiation reflects. Indeed, the *AFD* distribution suggests that appreciable lake-stream differentiation is widespread across the genome, thus questioning simple conclusions about the homogenizing effect of gene flow.

Together, these two empirical examples illustrate that using *F_ST_* as a differentiation metric can complicate the recognition and/or interpretation of patterns in population differentiation. The examples further serve as a general warning that in the face of real-world biological complexity, differentiation data alone are unlikely to allow inferring underlying evolutionary processes reliably—no matter what differentiation metric is applied. Combining differentiation data with biogeographic and demographic evidence, and with insights from additional population genetic analyses, will generally be required.

## 6. AFD—Recommendations for the Application

Given the appeal of *AFD* emerging from both conceptual considerations and empirical analysis, it becomes relevant to explore under what conditions this differentiation metric performs adequately. *AFD* is detached from theoretical assumptions or specific population genetic models, hence the only concerns when estimating population differentiation are that the samples represent the focal populations reliably—an issue of study design, and that sample sizes are large enough to estimate allele frequencies within each population reasonably precisely. To provide a point of reference for the latter criterion, I simulated the consequences of sampling a focal population pair with different intensities on estimates of *AFD*. Specifically, I modeled two populations with a precisely known allele frequency at a single SNP, choosing these frequencies such that the true parametric *AFD* value was 0, 0.05, 0.1, 0.25, or 0.5. Within each of these five scenarios of increasing population differentiation, I further considered up to five different MAF levels (0.025, 0.05, 0.125, 0.25, and 0.5) across the pool of the two samples, noting that with increasing differentiation, the range of possible MAFs decreases (e.g., with *AFD* = 0.5, the lowest possible parametric MAF is 0.25, see also Figure 1b). For each of the 19 total differentiation-by-MAF combinations considered, I then drew 10,000 replicate samples of equal size from each population, with sample size (i.e., the number of nucleotides) spanning the full range from 1 to 100, and calculated *AFD* between the populations for each replication (an analogous analysis based on *G_ST_* is presented as Figure S3).

**Figure 4:**
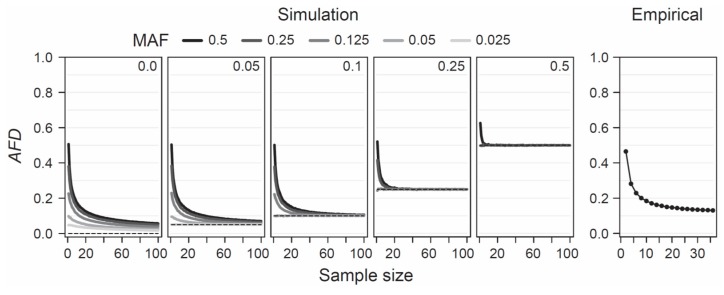
Sample size for *AFD*. Sensitivity of *AFD* to the size of the sample (number of nucleotides) taken from each population, explored by simulation (left) and using empirical population data (right). The simulations consider five different magnitudes of population differentiation (the true parametric differentiation is printed, and plotted as dashed line, inside each box), and up to five different MAF levels for each magnitude of differentiation (indicated by the gray shades of the lines). Note that with increasing differentiation, the possible MAF range becomes increasingly constrained. The lines show mean *AFD* across 10,000 replicate simulations for each sample size level. The empirical analysis shows mean *AFD* across the genome-wide SNPs from the lake-stream stickleback comparison shown in Figure 3b.

This analysis revealed that when sample size drops below around 20 nucleotides (corresponding to complete genotype data from 10 diploids) per population, *AFD* tends to become seriously biased upward (Figure 4, ‘Simulation’). This bias is most pronounced when both the true magnitude of population differentiation is low and the MAF is high. The reason becomes evident when we assume a SNP (e.g., alleles A and C) completely undifferentiated between two populations (parametric *AFD* = 0) and exhibiting a maximal MAF of 0.5 (i.e., both alleles occur in perfectly balanced proportion in both populations), as in the left bar plot of Figure 1a. If we randomly draw just two nucleotides from each population at this SNP, it is not unlikely (*p* = 0.125) to draw two identical alleles from one population and two opposite alleles from the other, and hence to observe complete differentiation (*AFD* = 1). Such overestimation, however, is not possible when the populations are undifferentiated but the MAF is minimal (i.e., both populations are fixed for the same allele; left bar plot in Figure 1b), or when differentiation is complete (populations fixed for opposite alleles; right bar plots in Figure 1a,b). As a general recommendation, sample sizes of 40–60 nucleotides per population (20–30 diploids) should thus suffice to achieve reasonably accurate estimates of population differentiation, irrespective of the true magnitude and the MAF.

To examine this recommendation with empirical data, I again used the SNP data set from the lake-stream stickleback population pair described above. I here assessed how genome-wide mean *AFD* (and *G_ST_*; Figure S3) changes when sampling both populations with a sample size ranging from 2 to 36. Across all sample sizes, I restricted the SNP panel to those represented by a least 36 nucleotides in each population, and I considered only SNPs displaying a MAF of 0.05 or greater across the population pool (a MAF threshold of 0.2 lead to the same conclusions; details not presented). This empirical exploration was in good agreement with the insights from the simulation analysis: the genome-wide mean *AFD* value became relatively stable with sample sizes of around 20–30 nucleotides per population (Figure 4, ‘Empirical’). I emphasize that all these conclusions regarding sample size are not specific to *AFD*; *F_ST_* shows a very similar sensitivity to sample size and to the associated precision in population allele frequency estimation, as presented in Figure S3a,b.

As a final methodological remark, I highlight that this article has so far considered only the situation in which the number of populations to be compared is exactly two. Although such pairwise population comparisons arguably represent the most common analytical situation, it should be noted that *F_ST_* statistics also permit estimating the overall genetic structure across a larger collection of populations. With *AFD* as a differentiation metric, this option is not available. A straightforward ad hoc solution, however, is to simply average multiple *AFD* values for SNPs or genome windows across the multiple population contrasts of interest [27].

## 7. Conclusions

The purpose of this note was to show that metrics of population differentiation used routinely in the analysis of genetic data—*F_ST_* statistics and related metrics—do not necessarily measure the quantities most meaningful in genetic and genomic research. As a point in favor of *F_ST_*, one may argue that its long tradition would promote the comparability of differentiation among studies [42]. This view, however, seems overly optimistic; *F_ST_* is highly contingent on the specific estimator, is sensitive to the MAF spectrum of the markers, and sometimes to imbalances in sample size. Combined with the general insensitivity across the differentiation range most relevant in many analytical situations—weak population differentiation—*F_ST_* falls short of being a reliable standard for measuring genetic differentiation. I argue that in many analytical contexts, the simple absolute allele frequency difference *AFD* will provide a sufficient, meaningful, and robust differentiation metric, thus promoting the discovery of patterns in differentiation, and their interpretation.

## Supplementary Materials

The following are available online at https://www.mdpi.com/2073-4425/10/4/308/s1, Figure S1: deviation of *Theta* and of the square-root of *G_ST_* from *AFD*, Figure S2: relationship between *G_ST_* and *AFD* for an empirical data set, Figure S3: sample size analysis for *G_ST_*, Analysis S1: worked example for the calculation of *AFD*.
