# Correction: Berner, D. Allele Frequency Difference *AFD*—An Intuitive Alternative to *F_ST_* for Quantifying Genetic Population Differentiation. *Genes* 2019, *10*, 308

**DOI:** 10.3390/genes10100810

**Published:** 2019-10-14

**Authors:** Daniel Berner

**Affiliations:** Department of Environmental Sciences, Zoology, University of Basel, Vesalgasse 1, CH-4051 Basel, Switzerland; daniel.berner@unibas.ch; Tel.: +41-(0)-612-0703-28

This note is to correct an error in my paper, concerning the Shannon differentiation metric (*D_Shannon_*) (Reference [43] in the paper). The paper states that *D_Shannon_* is undefined mathematically whenever one or both populations are monomorphic, that is, fixed for a single allele. Accordingly, the *D_Shannon_* curve in Figure 1a, showing population differentiation in relation to allele counts for the case in which the pooled minor allele frequency (MAF) is maximal, did not extend across the full range of allele counts; the rightmost data point reflecting complete population differentiation was missing. Moreover, *D_Shannon_* was completely missing in Figure 1b visualizing the continuum of allele frequency differentiation when the MAF is minimal (one population monomorphic across the entire allele count range).

The reason why *D_Shannon_* appeared undefined in these situations is that in monomorphic populations, the *p_i_ln(p_i_)* summand for the missing allele in the Shannon entropy formula (page 4 in the Supplementary Material to Reference [43]) will contain the logarithm of zero, which is negatively infinite. However, I overlooked that whenever the frequency of one allele is zero, the corresponding summand should be substituted by zero. Doing so produces a defined Shannon entropy value needed for the subsequent calculation of *D_Shannon_* according to the instructions on page 5 in the Supplementary Material to Reference [43]. Following this convention, *D_Shannon_* is indeed always defined and ranging between zero and one. The figure below is a copy of Figure 1 in the paper, but with *D_Shannon_* calculated by following the above convention.

I thank William Sherwin, Anne Chao, Lou Jost, and Peter Smouse for bringing this issue to my attention.

## Figures and Tables

**Corrected Figure 1:**
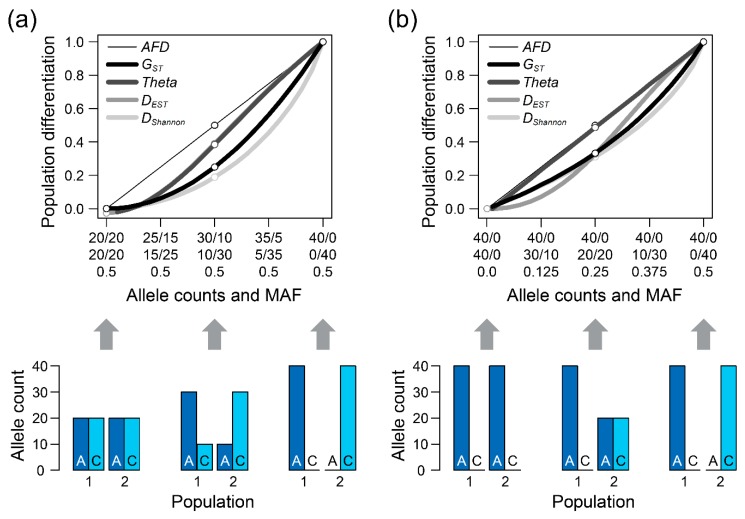
Population differentiation expressed by different metrics, including re-calculated Shannon differentiation (*D_Shannon_*). *G_ST_* and *Theta* were calculated according to the formulas (8) and (6) provided in Reference [28] in the paper. *D_EST_* was calculated using formula (13) in Reference [14]. All graphing conventions follow Figure 1 in the paper.

