# Supplementary material for: Allele Frequency Difference AFD–An Intuitive Alternative to FST for Quantifying Genetic Population Differentiation
Source: Genes (Basel). 2019 Apr 18;10(4):308. doi: 10.3390/genes10040308 (PMC6523497; doi:10.3390/genes10040308)
Supplement: Supplementary file 1 [file genes-10-00308-s001.pdf]

Supplementary Appendix to

Allele frequency difference *AFD* – an intuitive alternative to F_{ST} for quantifying genetic population differentiation

by
Daniel Berner

Department of Environmental Sciences, Zoology, University of Basel, Vesalgasse 1, CH-4051 Basel, Switzerland
daniel.berner@unibas.ch

Contents:

Fig. S1 Page 2
Fig. S2 Page 3
Fig. S3 Page 4
Analysis S1 Page 5

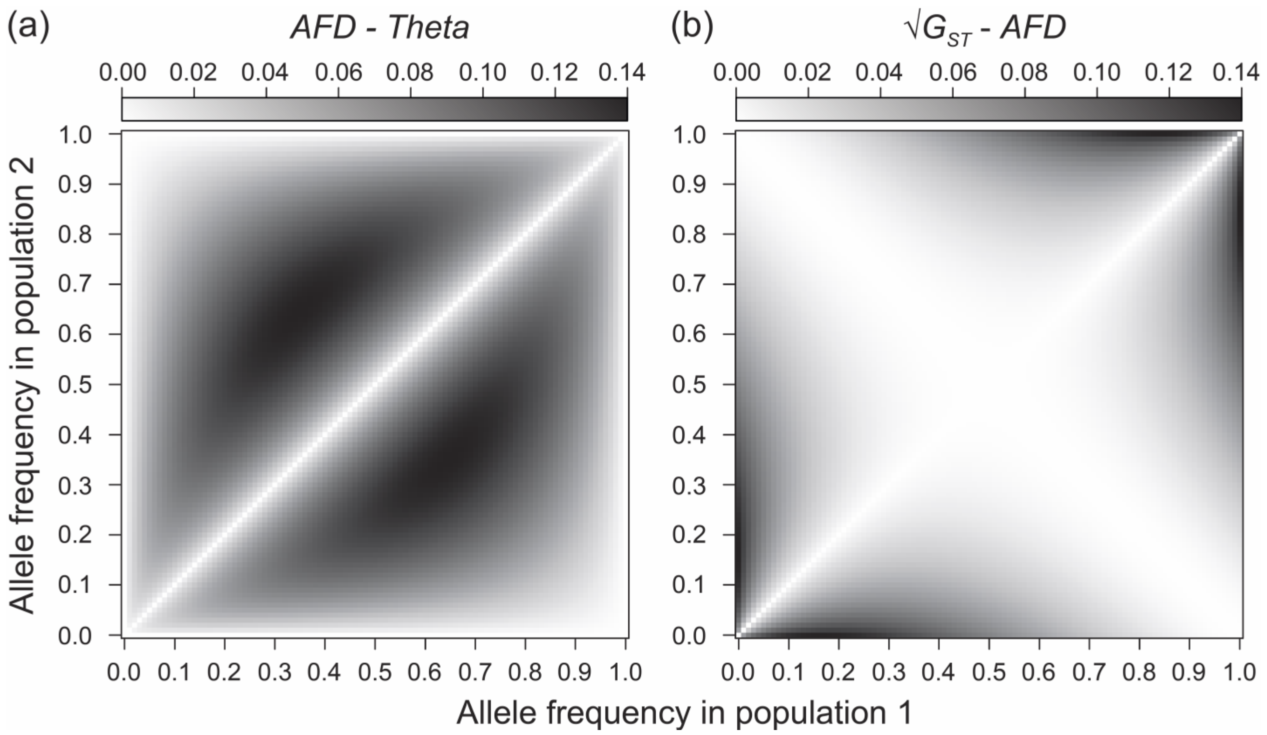

Fig. S1. Heatmaps showing the deviation of the F_{ST} estimator *Theta* (a) and of the square root of the F_{ST} *estimator* G_{ST} (b) from *AFD*, across combinations of exact allele frequencies between two populations at a single bi-allelic SNP. Allele frequencies (*X*- and *Y*-axes) refer to the same focal allele in both populations and were explored in increments of 0.01. For *Theta*, a sample size of 10,000 nucleotides per population was assumed throughout, thus approximating the theoretical values under infinite population size very closely (biologically more realistic sample sizes produced quantitatively very similar results). *Theta* coincides exactly with *AFD* when the populations display the same allele frequencies (absence of differentiation, *AFD* = 0; bottom-left to top-right diagonal), or when at least one population is monomorphic (edges of the graphic). Outside these domains, *Theta* underestimates *AFD*. The square root of G_{ST} coincides or approximates *AFD* across a broad range of allele frequency combinations, but overestimates *AFD* when one population is close to fixation for one allele and the magnitude of differentiation between the populations is low.

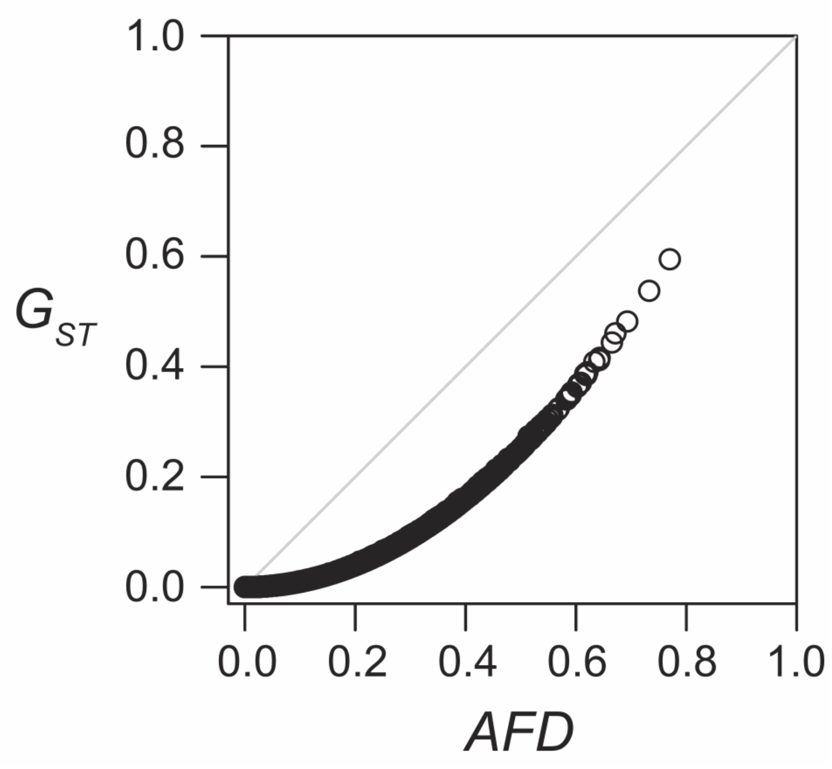

Fig. S2. F_{ST} estimator G_{ST} plotted against *AFD* across the 7282 single-nucleotide polymorphisms underlying the lake-stream stickleback population comparison presented in Figure 3b.

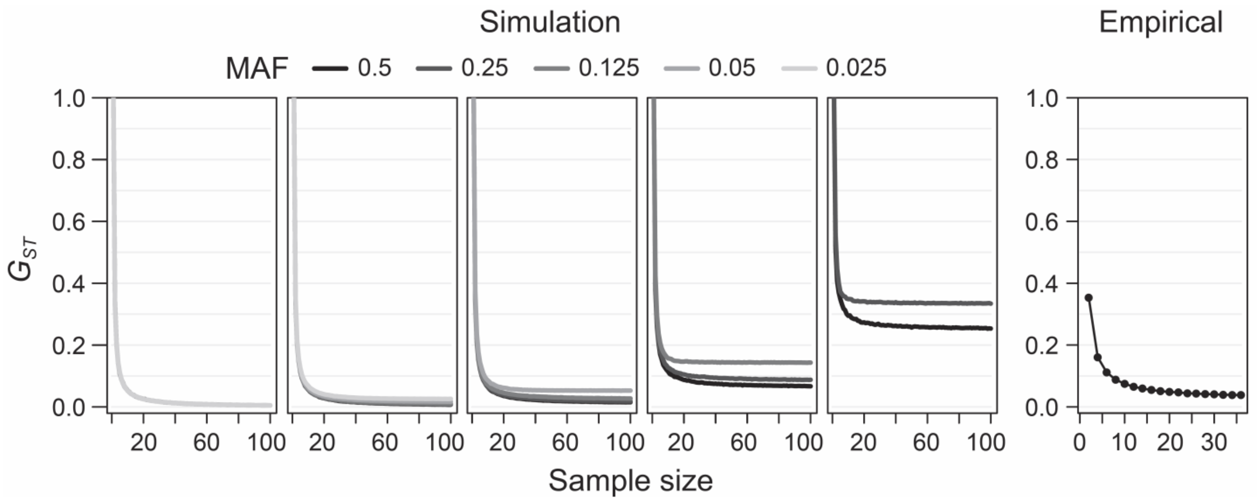

Fig. S3. Sensitivity of the F_{ST} estimator G_{ST} to the size of the sample (number of nucleotides) taken from each population, explored by simulation (left) and using empirical population data (right). The simulations consider the same five magnitudes of population differentiation as in Figure 4, increasing from no differentiation (left) to intermediate differentiation (right). The true parametric differentiation values, however, cannot easily be presented because they differ as a function of the MAF. As in Figure 4, up to five different MAF levels are considered for each magnitude of population differentiation (indicated by the gray shades of the lines; note that the lines overlap extensively when differentiation is low). The lines show mean G_{ST} across 10,000 replicate simulations for each sample size level. The empirical analysis shows mean G_{ST} across the genome-wide SNPs from the lake-stream stickleback comparison shown in Figure 3b.

Analysis S1. Worked example of *AFD* calculation for a bi-allelic single-nucleotide polymorphism (SNP; alleles *A* and *T*) and for a microsatellite marker with four di-nucleotide repeat length alleles. Sample sizes refer to the number of diploid individuals genotyped per population.

SNP

Population	Sample size	Allele count across all genotypes		Total count	Allele frequencies	
		A	***T***		f_{A1}	f_{T1}
1	14	18	10	28	18/28=0.643	10/28=0.357
		A	***T***		f_{A2}	f_{T2}
2	17	8	26	34	8/34=0.235	26/34=0.765
		Allele frequency differences:			$f_{A1} - f_{A2}$	$f_{T1} - f_{T2}$
					0.408	-0.408

To obtain the *AFD* statistic, the absolute values of the two allele frequency differences are added up, and this sum is divided by two, yielding ***AFD* = 0.408**. For a bi-allelic SNP, taking the absolute allele frequency difference calculated for a single allele will produce the same result (noting that the sign of the allele frequency difference may be of interest in some analytical context; for instance, when the focal allele is known to be the derived one).

Microsatellite

Population	Sample size	Allele count across all genotypes				Total count	Allele frequencies			
		**154**	**156**	**158**	**160**		f_{154_1}	f_{156_1}	f_{158_1}	f_{160_1}
1	20	7	23	10	0	40	7/40= 0.175	23/40= 0.575	10/40= 0.25	0/40= 0
		**154**	**156**	**158**	**160**		f_{154_2}	f_{156_2}	f_{158_2}	f_{160_2}
2	16	3	4	16	9	32	3/32= 0.094	4/32= 0.125	16/32= 0.5	9/32= 0.281
		Allele frequency differences					$f_{154\_1} - f_{154\_2}$	$f_{156\_1} - f_{156\_2}$	$f_{158\_1} - f_{158\_2}$	$f_{160\_1} - f_{160\_2}$
							0.081	0.45	-0.25	-0.281

The sum of the absolute allele frequency differences (0.081 + 0.45 + 0.25 + 0.281 = 1.062) divided by two yields ***AFD* = 0.531**.
